# Fertilizer source and application method influence sugarcane production and nutritional status

**DOI:** 10.3389/fpls.2023.1099589

**Published:** 2023-03-08

**Authors:** Sérgio Gustavo Quassi de Castro, Anderson Prates Coelho, Saulo Augusto Quassi de Castro, Thais Regina de Souza Chiachia, Rosilaine Araldi de Castro, Leandro Borges Lemos

**Affiliations:** ^1^ AgroQuatro-S Experimentation and Applied Agronomic Consultancy, Orlândia, São Paulo, Brazil; ^2^ School of Agricultural and Veterinarian Sciences, São Paulo State University (Unesp), Jaboticabal, São Paulo, Brazil; ^3^ Department of Soil Science, “Luiz de Queiroz” College of Agriculture, University of São Paulo, Piracicaba, São Paulo, Brazil; ^4^ Yara Brazil Fertilizers S/A, Porto Alegre, Rio Grande do Sul, Brazil

**Keywords:** liquid fertilizer, phosphorus, nitrogen, straw, potassium

## Abstract

**Introduction:**

The contrasting weather conditions throughout the sugarcane harvest period in south-central Brazil (April to November) influence fertilization management in sugarcane ratoon.

**Methods:**

Through field studies carried out over two cropping seasons, we aimed to compare the performance of sugarcane at sites harvested in the early and late periods of the harvest season as a function of fertilizer sources associated with application methods. The design used in each site was a randomized block in a 2 x 3 factorial scheme; the first factor consisted of fertilizer sources (solid and liquid), and the second factor consisted of application methods (above the straw, under the straw, and incorporated into the middle of the sugarcane row).

**Results:**

The fertilizer source and application method interacted at the site harvested in the early period of the sugarcane harvest season. Overall, the highest sugarcane stalk and sugar yields at this site were obtained with the incorporated application applying liquid fertilizer and under straw applying solid fertilizer, with increments of up to 33%. For the site harvested in the late period of the sugarcane harvest season, the liquid fertilizer promoted a 25% higher sugarcane stalk yield compared to the solid fertilizer in the crop season with low rainfall in the spring, while in the crop season with normal rainfall, there were no differences between treatments.

**Discussion:**

This demonstrates the importance of defining fertilization management in sugarcane as a function of harvest time, thereby promoting greater sustainability in the production system

## 1 Introduction

Sugarcane is a crop with high relevance worldwide, being the main raw material for sugar production (Yang et al., 2021) and the second raw material for ethanol production (Otto et al., 2022a), in addition to having great potential for bioenergy generation (Cherubin et al., 2018). Brazil is the largest sugarcane producer, with 9 million hectares, and sugarcane is the third-most planted crop in the country. The south-central region of Brazil is the main sugarcane production region, presenting the largest cultivation area (85%), especially in the state of São Paulo (4.4 million hectares) (Conab, 2022). Sugarcane is a semi-perennial crop in which soil fertilization in plant regrowth (i.e., sugarcane ratoon) is usually done 60 days after the crop has been harvested. The main nutrients applied to the soil are nitrogen (N), potassium (K), and phosphorus (P) due to respectively high removal of these nutrients by the plant (Cherubin et al., 2019).

The sugarcane harvest season in the south-central region of Brazil extends from the beginning of autumn (March/April) to the end of spring (November) (Otto et al., 2016). Autumn and winter are dry and cold seasons in the south-central region of Brazil, leading to a water deficit in the soil and limiting nutrient availability to the plant (Thorburn et al., 2017; Corrêa et al., 2019). Thus, soil NPK fertilization in sugarcane ratoons carried out in unfavorable weather conditions (i.e., drought season) may lead to the absence of production gain (Su et al., 2012; Otto et al., 2016), lower economic return from fertilization (Castro et al., 2019), and a decrease in nutrient accumulation and use efficiency by the plants (Fageria and Baligar, 2005; Dinh et al., 2017; Oliveira Filho et al., 2021), which might increase environmental losses (Bowles et al., 2018).

These effects are intensified by the mechanized sugarcane harvesting system. In the mechanized sugarcane harvesting system, a thick layer of straw is left on the soil surface, 10 to 20 Mg ha^−1^ (Carvalho et al., 2017; Bordonal et al., 2018; Silva et al., 2019). Once soil fertilization is commonly band applied onto the soil surface (Castro et al., 2017), the straw layer acts as a physical barrier, interfering with the contact of the fertilizers and the soil and affecting the availability of nutrients to plants.

To increase the nutrient availability, accumulation, and use efficiency of plants in the current sugarcane crop system, new fertilization management must be adopted. They must fit the program of good practices for the efficient use of fertilizers (GMPs), i.e., the four R’s policy (4R’s). The 4R’s consist of the application of the right source of the nutrient, at the right rate, using the right application method, at the right time (Bruulsema et al., 2009). These actions provide for agricultural crop (e.g., sugarcane) improvements in agronomic management worldwide, agreeing with environmental, social, and governance (ESG) policies (Carvalho et al., 2021).

The most studied 4R’s action in sugarcane consists of the correct rate of nutrients. This variable is highly influenced by the application time once weather conditions mediate the sugarcane response to fertilization. Recent studies indicate that the adjustment of the fertilizer rate according to the sugarcane harvest season (i.e., autumn, winter, or spring) promotes production gains (Castro et al., 2022) and increases nutrient use efficiency by the plant (Bowles et al., 2018; Congreves et al., 2021; Farzadfar et al., 2021). These increments are obtained when soil fertilization has been carried out under conditions of adequately high soil moisture, which favors nutrient availability for plant uptake (Castro et al., 2022; Otto et al., 2022b). However, under excessively high soil moisture and water logging conditions, nutrient losses might increase due to denitrification and leaching (Otto et al., 2016; Bowles et al., 2018).

Fertilizer application method studies have been conducted in recent years using the sugarcane harvest system. As it is necessary to trespass the sugarcane straw layer barrier to apply the fertilizer onto or into the soil, i.e., closer to plant roots, new machines have been developed (Silva et al., 2017). Previous studies have demonstrated that adjusting the fertilizer application method to apply N fertilizer incorporated into the soil in the middle of or next to the sugarcane crop row increases plant biomass production (Castro et al., 2017; Borges et al., 2019). More gains might be obtained when using this method to apply NPK fertilizer due to greater N, P, and K availability and uptake by the plant; however, this has not been well investigated.

The last 4R’s action is the choice of the right nutrient source. This is especially important for sugarcane because the subproducts of sugarcane processing are commonly applied in the field. Studies have indicated that these residues, e.g., filter cake (Soltangheisi et al., 2019) and vinasse (Cherubin et al., 2019), green manure (Tenelli et al., 2019), and organic fertilizers (Gutiérrez-Miceli et al., 2017; Bautista et al., 2019), partly meet the nutritional quality requirements of crop-complementing inorganic fertilizers. When discussing the sources of inorganic fertilizers to be applied, it is common to think of the different solid fertilizers that can be used. Studies have focused on evaluating solid fertilizer solubility, losses to the environment, and effects on sugarcane growth. However, as soil fertilization might be carried out during the drought season, the use of liquid fertilizer seems more adequate to promote great nutrient accumulation by the plant and sugarcane growth (Ullah et al., 2019) due to the soil moistening promoted by the volume of liquid fertilizer applied (800 to 1000 L ha^−1^). This hypothesis is based on experiments that have investigated sugarcane fertilization, which promotes gains in biomass production and in the technological quality of the raw material (Rhein et al., 2016; Gutiérrez-Miceli et al., 2017; Dalri et al., 2021). To the best of our knowledge, no published study has investigated the use of liquid NPK fertilizer compared to the solid source on sugarcane biomass production and N, P, and K accumulation.

Therefore, investigations on the effects of fertilizer sources (i.e., liquid or solid) and application methods in sugarcane harvested at different moments throughout the cropping season (i.e., harvested in autumn/winter and spring) are needed. They are essential for the recommendation of more accurate NPK fertilization management, aiming to increase biomass production and nutrient accumulation by the plant. The present study hypothesized that the fertilizer source and application method interact with each other and result in specific recommendations for sugarcane ratoon sites as a function of harvest time. The objective of this study was to evaluate and compare the performance of sugarcane ratoon sites (i.e., site harvested at the beginning [autumn/winter] or end [spring] of the harvest season) as a function of the application of liquid and solid fertilizers associated with the application method (i.e., above the straw layer [ASt], under the straw [USt], and incorporated into the soil in the middle of the sugarcane cultivation row [SI]).

## 2 Materials and methods

### 2.1 Field description

This research project was carried out at AgroQuatro-S Experimentation and Applied Agronomic Consultancy, located in Sales Oliveira, São Paulo state, Brazil (20°51’35’’S, 47°56’24’’W and altitude of 575 m). Two experimental sites (sugarcane ratoon cycles) were conducted during the two sugarcane cropping seasons (i.e., 2019/2020 and 2020/2021). The first site (site 1 – early period of the harvest season) was harvested in June (autumn/winter), and the second site (site 2 – late period of the harvest season) was harvested in September/October (spring). The decision to carry out the experiment considering sites with different harvest season periods was related to the focus of evaluating sugarcane’s agronomic response as a function of the harvest season period, which might influence the sugarcane response (i.e., aboveground biomass production and N, P, and K accumulation by the plant). Once the harvest seasons began in March/April and ended in October/November in the south-central region of Brazil (the largest sugarcane producer region in the country) (Carvalho et al., 2019), these sites were chosen. The sugarcane varieties adopted in sites 1 and 2 were RB966928 and RB965902, respectively.

Before setting up the experiment, i.e., after sugarcane had been harvested in 2019, soil was sampled at both sites at four depths (0–0.2, 0.2–0.4, 0.4–0.6, and 0.6–0.8 m). The soil samples were chemically and physically characterized. The soil pH was determined in a 0.01 M CaCl_2_ solution, and soil organic matter was determined using dichromate oxidation. Sulfur was extracted using calcium phosphate (0.01 M), and phosphorus, potassium, calcium, and magnesium were extracted following the resin method (1 M NaHCO_3_ at pH 8.5). H + Al was determined using the buffer SMP method, and Al was determined with KCl extraction following analysis in atomic absorption spectrometry or spectrophotometry (Raij et al., 2001). The soil was classified as Eutrudox (Soil Survey Staff, 2014), with a very clayey texture in both areas (**Table 1**).

**Table 1:** Soil chemical characterization and physical attributes evaluated, before the treatment implementation in site 1 and in site 2.

Layer	pH	SOM	S	P	K	Ca	Mg	H+Al	Al	SB	CEC	V	Sand	Clay	Silt	Texture
m	CaCl_2_	g dm^-3^	-mg dm^-3^-		————– mmol_c_ dm^-3^ ————–							%	—— g kg^-1^ ——			
Site 1 - Early period of the harvest season																
0-0.2	5.2	40	9	10	1.4	43	8	47	0	52	99	53	173	496	331	C
0.2-0.4	5.7	34	8	8	0.6	47	8	34	0	56	90	62	172	543	285	C
0.4-0.6	6.1	23	9	7	0.4	36	6	22	0	42	64	66	157	582	261	C
0.6-0.8	6.3	19	9	7	0.4	32	5	19	0	56	56	67	157	608	235	VC
Site 2 - Late period of the harvest season																
0-0.2	5.4	32	13	9	1.1	41	13	34	0	55	89	62	227	490	283	C
0.2-0.4	5.7	24	5	8	0.5	40	10	26	0	51	77	66	215	539	246	C
0.4-0.6	6.1	19	7	6	0.4	26	7	19	0	33	52	64	154	623	223	VC
0.6-0.8	6.3	15	11	4	0.3	23	6	17	0	29	46	64	135	613	252	VC

SOM, Soil Organic Matter; SB, Sum of Bases; CEC, Cation Exchange Capacity; V, Base Saturation; C, clayey; VC, very clayey.

### 2.2 Experimental design

**Figure 1:**
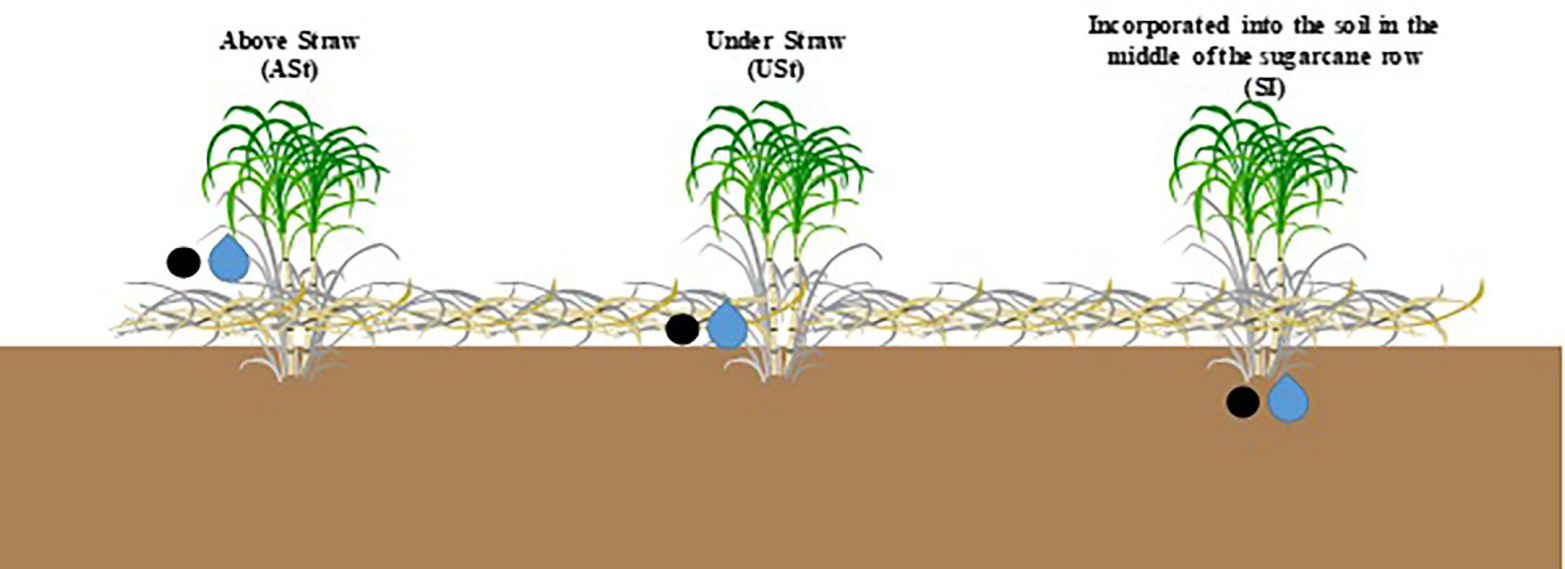
Fertilizer application methods evaluated in the experiment. Fertilizer sources, i.e., solid and liquid, are shown by the black circle and blue drop, respectively.

The experimental design adopted at both sites in the two cropping seasons was a randomized block in a 2 x 3 factorial scheme with four replicates. The main factor was the fertilizer source (i.e., liquid or solid), and the second factor was the fertilizer application method (i.e., above straw [ASt], under straw [Ust], and incorporated into the soil in the middle of the sugarcane row [SI]) (**Figure 1**). Each plot consisted of five sugarcane rows, 10 m in length. The sugarcane interrow spacing in both experimental fields was 1.5 m.

The treatments were installed in 2019 and reinstalled in the same position in 2020, as the experiments were carried out during two cropping seasons. At site 1 (i.e., early harvest season), sugarcane was harvested in June; therefore, treatments were applied in July 2019 (first crop season) and July 2020 (second crop season). At site 2 (i.e., late harvest season), sugarcane was harvested in November, and treatments were applied in December 2019 (first crop season) and December 2020 (second crop season). The treatments were evaluated before the sugarcane harvest, i.e., June 2020 and June 2021 at site 1 and November 2020 and September 2021 at site 2. In the second crop season (2020/2021), harvesting was performed in September at site 2 due to adverse weather conditions. The chronological sequence of the experiment is presented in **Supplementary Figure 1** to show the timing of agronomic operations.

The nutrient rates applied at both sites and in both crop seasons were 105, 22, and 87 kg ha^−1^ of N, P, and K, respectively. To apply these rates, 552 kg ha^−1^ of solid fertilizer 19-09-19 and 880 L ha^−1^ of liquid fertilizer 12-06-12 (density of 1.326 g cm³) were applied in their corresponding plots. NPK fertilizer was applied manually in each sugarcane row in the plot according to the respective application method and fertilizer source. In the USt application method, sugarcane straw was manually removed, and fertilizer sources (i.e., liquid or solid) were applied onto the soil, followed by the relocation of the straw onto the soil. In the SI application method, a specific machine opened the furrow in the middle of the sugarcane row, followed by the manual application of fertilizer sources into the furrow. In the ASt application, fertilizer sources were deposited above the sugarcane straw. These application methods are commonly used in sugarcane fields (Franco et al., 2017). Prior to application, the fertilizer rate for each plot line was calculated using a digital balance (solid fertilizer), beakers, and volumetric flask (liquid fertilizer).

Micronutrients (boron [B], zinc [Zn], and molybdenum [Mo]) were sprayed on the sugarcane canopy twice throughout crop development. The first application was carried out in December, and the second application was carried out in February in each experimental crop season. The micronutrient rates adopted in each foliar application were 136 g ha^−1^ of B, 700 g ha^−1^ of Zn, and 23 g ha^−1^ of Mo; the flow rate used was 150 L ha^−1^, using CO_2_ spraying.

### 2.3 Parameters evaluated

Sugarcane parameters (i.e., aboveground biomass production and N, P, and K accumulation by the plant, and the technological quality of the raw material) were evaluated in the middle of each plot at both sites in both crop seasons. Two meters located in the center of the three middle crop rows (i.e., evaluation area) of each plot were used for data collection. These evaluations were carried out before mechanical sugarcane harvest, i.e., June 2020 and June 2021 at site 1 and November 2020 and September 2021 at site 2.

First, the sugarcane plant population was evaluated (number of stalks per meter) in the evaluation area of each plot. Afterward, aboveground biomass production was determined; sugarcane plants were subdivided into three plant tissues (stalks, tops, and dry leaves), which were weighted to determine the plant tissues’ fresh mass. Using the fresh mass, plant tissue production per hectare (Mg ha^−1^) was calculated by considering 6667 m of sugarcane row in 1 ha^−1^. In this evaluation, 10 stalks of sugarcane were collected to evaluate the technological quality of raw material represented by the percentage of sucrose (PC [%]), soluble solid (Brix [%]), and fiber (Fiber [%]), following the methodology proposed by Fernandes (2003). In addition, the total recoverable sugar (TRS [kg TRS Mg^−1^ of stalk]) was also determined, and the sugar yield based on stalk production (Mg TRS ha^−1^) was calculated.

Sub-samples of each plant tissue were obtained after they had been ground in a forage grinder. The sub-samples of dry leaves, tops, and stalks were dried in an oven with air circulation at 65°C until achieving a constant weight. The dried sub-samples of each plant tissue were ground in a Wiley mill coupled with a 0.5 mm sieve, following N, P, and K quantification. They were determined according to the methodology described by Bataglia et al. (1983). The total nitrogen content was determined using wet digestion with sulfuric acid, followed by determination using the micro-Kjeldahl method. Phosphorus and potassium were determined using digestion with nitric-perchloric acid, followed by determination with atomic absorption spectrometry. N, P, and K accumulation by the sugarcane plant (kg ha^−1^) were determined by summing the accumulation of these nutrients in each plant tissue. Nutrient accumulation in each plant tissue was determined by multiplying the nutrient content (g kg^−1^) by the plant tissue dry mass (Mg ha^−1^).

### 2.4 Weather conditions

**Figure 2:**
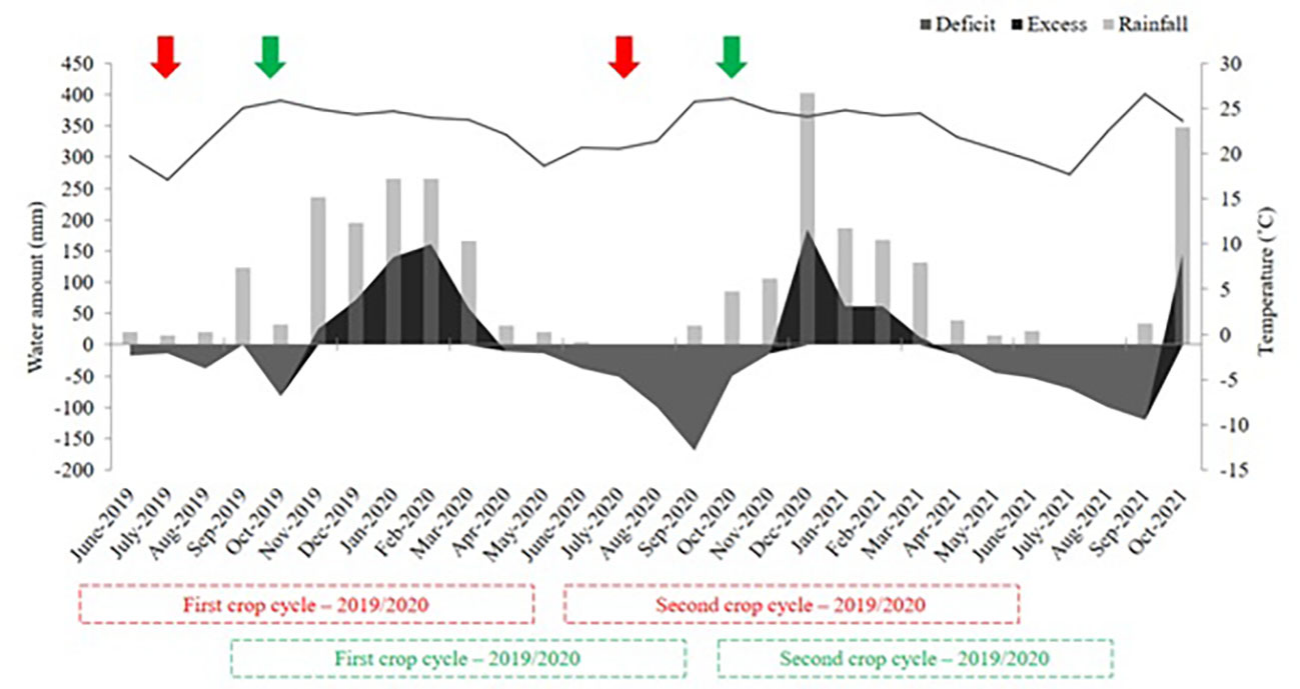
Water balance over the experimental period. Arrows indicate the fertilization time in the site 1 (early-harvest site [red]) and in the site 2 (late-harvest site [green]) in sugarcane experimental fields.

Over the experimental period, weather conditions (e.g., rainfall and temperature) were monitored using an automatic weather station installed closer to the sites. Using these weather parameters, the water balance (**Figure 2**) was calculated according to the methodology described by Thornthwaite and Mather (1955).

At site 1 (early harvest area), the total rainfall accumulated during the first crop season (2019/2020) was 1397 mm, while water excess and deficit were 458 and −215 mm, respectively. In the second crop season (2020/2021), the total rainfall accumulated and water excess and deficit were 1188, 321, and −500 mm, respectively. At site 2 (late harvest area), the total rainfall accumulated in the first crop season (2019/2020) was 1335 mm, while water excess and deficit were 455 and −517 mm, respectively. In the second crop season (2020/2021), the total accumulated rainfall and water excess and deficit were 1106, 320, and −420 mm, respectively.

### 2.5 Statistical analysis

All data were subjected to analysis of variance (F test) at a 5% significance level. When necessary, according to the principles of data normality, the Shapiro–Wilk test (Royston, 1995) was used, and according to homoscedasticity of variances, the Levene test (Gastwirth et al., 2009) was used; otherwise, the means of the variables were compared using Tukey’s test (p<0.05). For each crop season, the results were subjected to an individual analysis of variance. Subsequently, for the comparison between the crop seasons considering each site, a joint analysis of the experiments was performed when the ratio between the highest and lowest residual mean square (RMS) of each variable did not exceed a value of seven (Banzatto and Kronka, 2006). Univariate statistical analyses were performed using AgroEstat software (Barbosa and Maldonado, 2015).

## 3 Results

### 3.1 Early harvest site

For the site harvested at the beginning of the sugarcane harvest season (site 1), the fertilizer source did not affect any of the variables in the first crop season, while the application method interfered with the sugarcane plant population (stalks m^−1^), stalk and sugar yield, and N and P accumulation (**Table 2**). In the second crop season, the fertilizer source did not affect any of the variables, while the application method interfered with N accumulation. There was an interaction between fertilizer source and application method in the two crop seasons for stalk and sugar yield. When comparing the crop seasons, the second crop season had the highest values for plant population, Brix, Fiber, PC, TRS, and N and P accumulation.

**Table 2:** Summary of the analysis variance for the production, technological and nutritional variables of sugarcane in the site 1 (early-harvest site) as a function of fertilizer sources and application methods in the two crop seasons.

Source of variation	Stalks n° m^-1^	StY t ha^-1^	Brix %	Fiber %	PC %	TRS kg t^-1^	SY t ha^-1^	N kg ha^-1^	P kg ha^-1^	K kg ha^-1^
	————————————–p-value - 2020————————————–									
Fertilizer sources (F)	0.87	0.64	0.22	0.75	0.18	0.19	0.66	0.88	0.68	0.75
Application Methods (MA)	0.01	<0.01	0.60	0.41	0.58	0.59	<0.01	0.02	0.01	0.14
F x MA	0.10	<0.01	0.79	0.89	0.64	0.69	<0.01	0.16	0.06	0.35
CV (%)	10.9	5.9	3.6	2.9	4.4	4.1	7.0	14.4	16.4	24.5
	————————————–p-value - 2021————————————–									
Fertilizer sources (F)	0.63	0.35	0.51	0.55	0.54	0.59	0.47	0.35	0.19	0.33
Application Methods (MA)	0.19	0.49	0.34	0.17	0.47	0.50	0.46	0.02	0.72	0.16
F x MA	0.48	<0.01	0.39	0.43	0.69	0.62	<0.01	0.30	0.29	0.10
CV (%)	10.8	10.6	2.1	2.5	2.6	2.5	12.3	12.5	13.1	13.8
Joint Analysis										
Highest RMS/Lowest RMS	2.36	3.48	2.71	1.12	2.76	2.57	3.55	1.22	1.09	3.10
	p-value									
Treatments (T)	0.12	0.04	0.90	0.77	0.89	0.89	0.03	<0.01	0.10	0.04
Crops Seasons (Y)	<0.01	0.27	<0.01	<0.01	0.04	0.03	0.07	0.04	<0.01	0.85
T x Y	0.50	0.12	0.36	0.38	0.36	0.40	0.36	0.97	0.38	0.80
2019/2020	13.9 b	113	20.6 b	11.0 b	16.1 b	158.8 b	17.9	135 b	15.5 b	177
2020/2021	21.6 a	117	21.5 a	11.8 a	16.6 a	163.6 a	19.2	141 a	18.6 a	178

Stalks, Plant population; StY, stalk yield; PC, Pol of cane; TRS, Total recoverable sugar; SY, sugar yield; CV, coefficient of variation.

**Figure 3:**
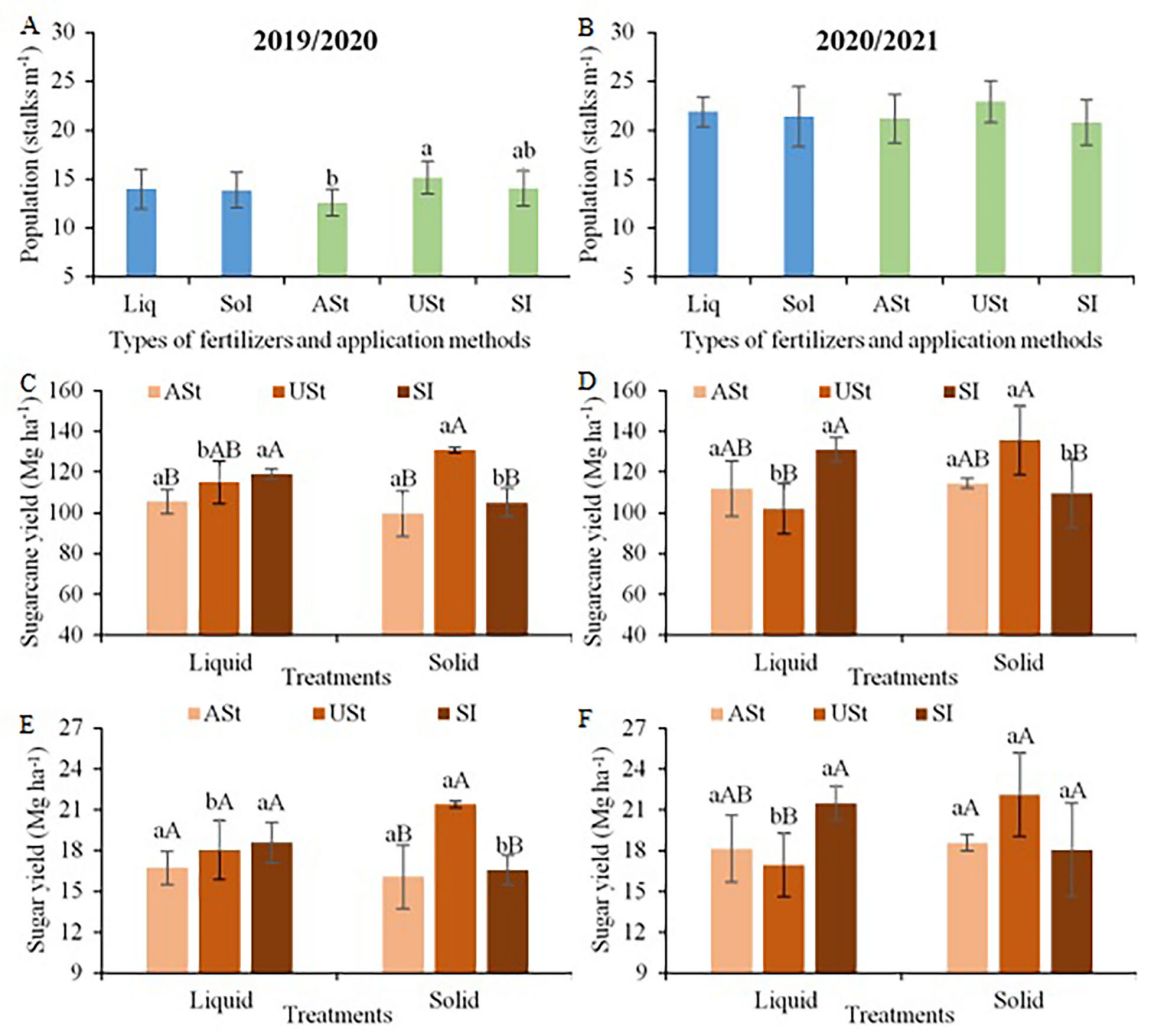
Comparison of means for plant population **(A, B)**, sugarcane yield **(C, D)** and sugar yield **(E, F)** in the site 1 (early-harvest site) as a function of fertilizer source and application method. Lowercase letters compare the fertilizer source for each application method and uppercase letters compare the application method for each fertilizer source. Liq, Liquid fertilizer; Sol, Solid fertilizer; ASt, Application above straw; USt, Application under straw; SI, Application incorporated into the soil.

The stalk population (stalks m^−1^) showed increments of 21% when fertilizer was applied under straw (USt) in the first crop season (**Figure 3A**), with no difference in the second crop season (**Figure 3B**). The application of liquid fertilizer incorporated into the soil in the middle of the sugarcane row (SI) promoted higher values of stalk yield compared to the same application method applying solid fertilizer in the two crop seasons (**Figures 3C, D**), with increments of up to 20%. However, the USt application of solid fertilizer promoted increases in stalk yield compared to the same application method applying liquid fertilizer in the two crop seasons, with a superiority of up to 33%. For the application above the sugarcane straw (ASt), there were no differences between the fertilizer sources. When comparing the application methods for each fertilizer source, the SI application promoted yield up to 28% higher for the liquid fertilizer in the two crop seasons, while for the solid fertilizer, the highest values were obtained with the USt application, with increments of up to 31% compared to the other application methods.

Regarding sugar yield, the SI application of liquid fertilizer showed a trend of higher values compared to the USt application, with statistical differences only in the second crop season (**Figures 3E, F**). For solid fertilizer, the USt application showed a trend of higher values compared to the other application methods, with differences only in the first crop season. Comparing the application methods for each type of fertilizer, the USt application of solid fertilizer promoted higher values of sugar yield compared to the same application method applying liquid fertilizer, with differences of up to 30%. In addition, the SI application of liquid fertilizer tended to show higher means compared to the same application method applying solid fertilizer, with differences only for the second crop season (+19%).

**Figure 4:**
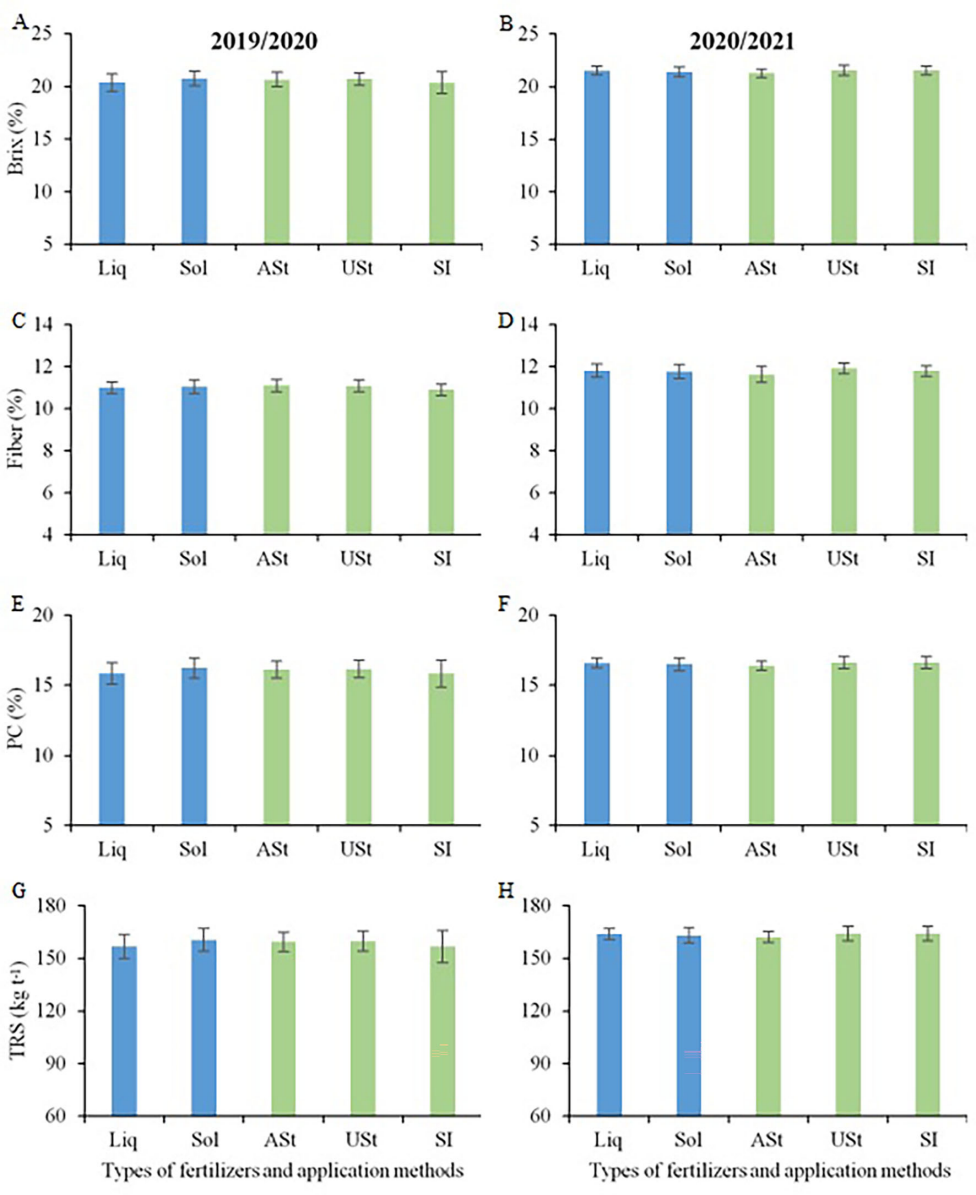
Comparison of means for Brix **(A, B)**, Fiber content **(C, D)**, Pol of cane - PC **(E, F)** and total recoverable sugar – TRS **(G, H)** of sugarcane in the site 1 (early-harvest site) as a function of the fertilizer source and application method. Liq, Liquid fertilizer; Sol, Solid fertilizer; ASt, Application above straw; USt, Application under straw; SI, Application incorporated into the soil.

No technological variable for sugarcane was influenced by the study factors in the early harvest area (**Figure 4**). In the two crop seasons, Brix values ranged from 20.4 to 21.6%, fiber from 10.9 to 11.9%, PC from 15.8 to 16.6%, and TRS from 157 to 164 kg TRS Mg^−1^ of stalk.

**Figure 5:**
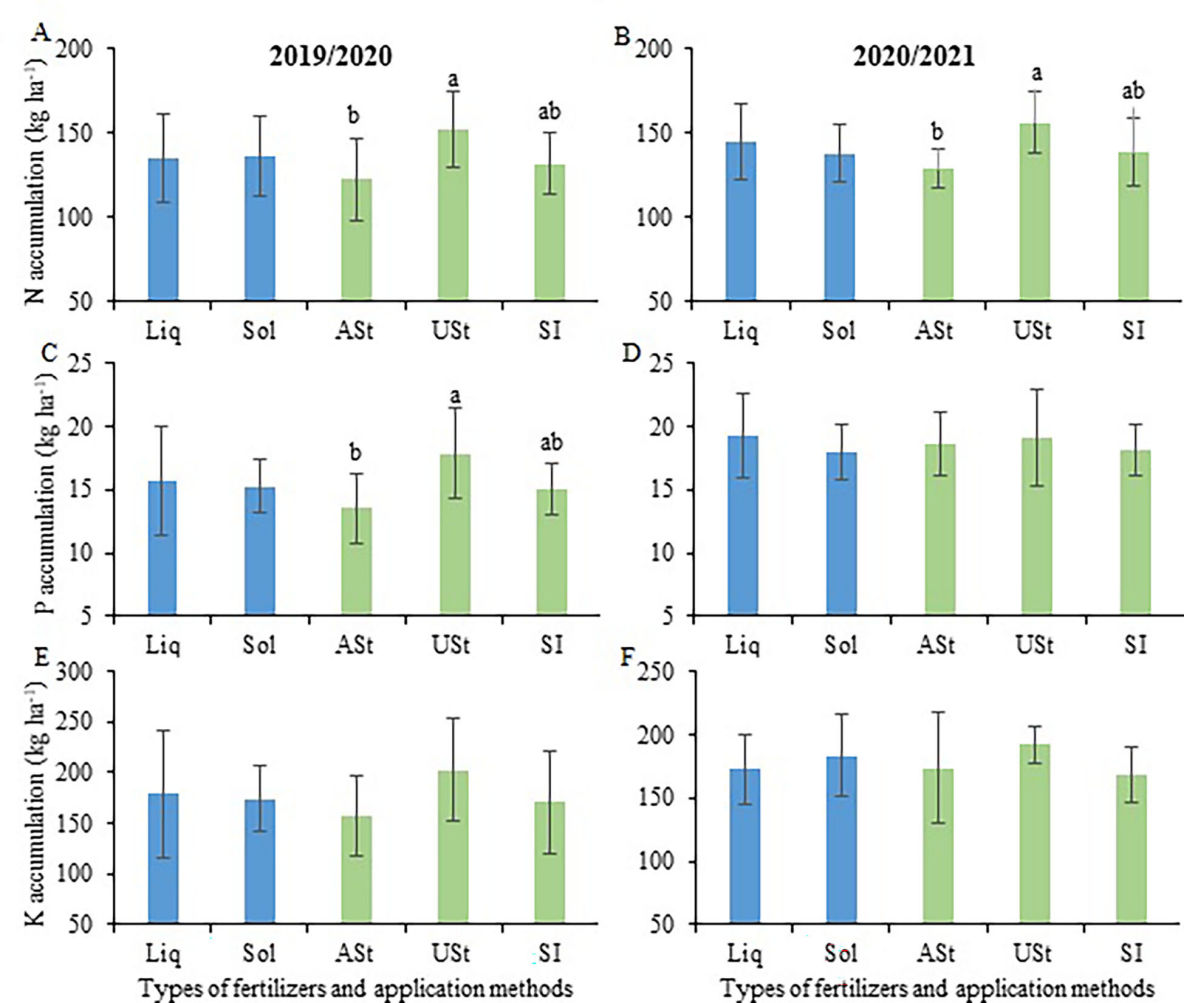
Comparison of means for the total accumulation of N **(A, B)**, P **(C, D)** and K **(E, F)** of sugarcane in the site 1 (early-harvest site) as a function of the fertilizer source and application method. Liq, Liquid fertilizer; Sol, Solid fertilizer; ASt, Application above straw; USt, Application under straw; SI, Application incorporated into the soil. Lowercase letters compare fertilizer application methods.

The nutrient levels (N-P-K) obtained in each plant fraction (i.e., stalk, tops, and dry leaves) are shown in **Supplementary Tables 1–3**. Overall, there were few differences between treatments within each crop season. The P content in the stalk was higher in 2019/2020, and the P content in the tops plant was higher in 2020/2021. The N accumulation was higher in the USt application method than in the ASt application method, with a value of up to 24% higher (**Figures 5A, B**). This observation was made for P accumulation in the first crop season, with a value 31% higher (**Figure 5C**). None of the study factors influenced K accumulation by sugarcane, with values ranging from 157 to 202 kg K ha^−1^ in both crop seasons.

### 3.2 Late harvest site

At the site harvested at the end of the sugarcane season (site 2), the fertilizer source affected the plant population in the first crop season, while the application method interfered with K accumulation (**Table 3**). In the second crop season, the fertilizer source affected the stalk and sugar yield, while the application method interfered with N accumulation. There was no interaction between fertilizer source and application method for any of the variables in the two crop seasons. The first crop season showed a higher stalk yield (+29%) and sugar yield (+28%), while the second crop season had the highest values for Brix, fiber, and N, P, and K accumulation. In addition, the interaction between treatments and crop seasons was significant for both stalk and sugar yield.

**Table 3:** Summary of the analysis variance for the production, technological and nutritional variables of sugarcane in the site 2 (late-harvest site) as a function of fertilizer sources and application methods in the two crop seasons.

Source of variation	Stalks n° m^-1^	StY t ha^-1^	Brix %	Fiber %	PC %	TRS kg t^-1^	SY t ha^-1^	N kg ha^-1^	P kg ha^-1^	K kg ha^-1^
	————————————–p-value - 2020————————————–									
Fertilizer sources (F)	0.03	0.13	0.65	0.47	0.65	0.66	0.15	0.09	0.73	0.36
Application Methods (MA)	0.31	0.14	0.32	0.10	0.33	0.35	0.31	0.49	0.30	<0.01
F x MA	0.58	0.87	0.40	0.05	0.56	0.56	0.99	0.56	0.21	0.20
CV (%)	11.5	11.7	3.9	2.5	4.8	4.6	13.6	20.9	16.9	16.9
	————————————–p-value - 2021————————————–									
Fertilizer sources (F)	0.49	<0.01	0.28	0.82	0.27	0.27	<0.01	0.14	0.40	0.22
Application Methods (MA)	0.15	0.10	0.75	0.17	0.96	0.95	0.10	<0.01	0.45	0.17
F x MA	0.61	0.19	0.59	0.97	0.46	0.47	0.17	0.45	0.65	0.17
CV (%)	15.0	11.1	2.6	2.9	2.9	2.8	11.6	10.7	19.0	16.7
Joint Analysis										
Highest RMS/Lowest RMS	1.98	1.84	2.16	1.58	2.65	2.61	2.25	2.09	4.24	1.65
	p-value									
Treatments (T)	0.93	0.97	0.37	0.78	0.19	0.19	0.97	0.30	0.49	0.24
Crop Seasons (Y)	0.26	0.02	<0.01	<0.01	0.24	0.18	0.03	<0.01	<0.01	0.01
T x Y	0.07	<0.01	0.63	0.08	0.82	0.82	<0.01	0.05	0.58	0.12
2019/2020	12.3	107 a	22.9 b	11.1 b	18.1	178	19.2 a	110.1 b	11.2 b	134.9 b
2020/2021	13.3	83 b	23.7 a	11.9 a	18.3	180	15.0 b	149.1 a	20.6 a	175.0 a

Stalks, plant population; StY, stalk yield; PC, Pol of cane; TRS: Total recoverable sugar; SY, sugar yield; CV, coefficient of variation.

**Figure 6:**
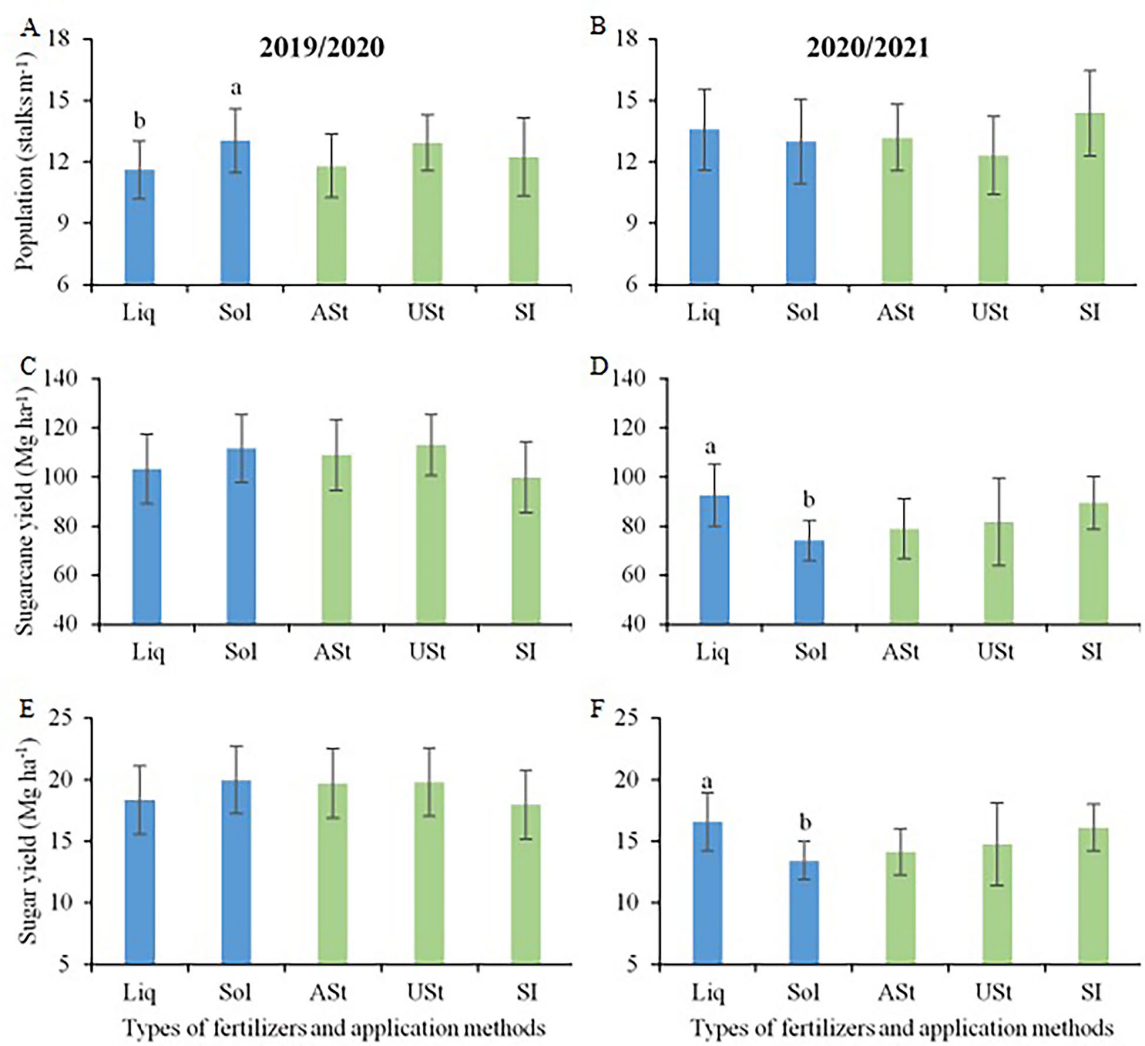
Comparison of means for plant population **(A, B)**, sugarcane yield **(C, D)** and sugar yield **(E, F)** in the site 2 (late-harvest site) as a function of the fertilizer source and application method. Liq, Liquid fertilizer; Sol, Solid fertilizer; ASt, Application above straw; USt, Application under straw; SI, Application incorporated into the soil. Lowercase letters compare fertilizer sources.

Applying solid fertilizer increments (+12%) in the stalk population was promoted in the first crop season compared to liquid fertilizer (**Figure 6A**). In the second crop season, although there was a greater amplitude in the population (12 to 14 stalks m^−1^), there was no difference according to the fertilizer source (solid or liquid) and application method (St, USt, or SI) (**Figure 6B**). The application of liquid fertilizer promoted gains in stalk yield (+25%) when compared to solid fertilizer in the second crop season (**Figure 6D**). Moreover, the fertilizer application method did not affect the stalk yield. The same trend was observed for sugar yield, in which liquid fertilizer generated a higher value (+23%) compared to solid fertilizer in the second crop season (**Figure 6F**).

**Figure 7:**
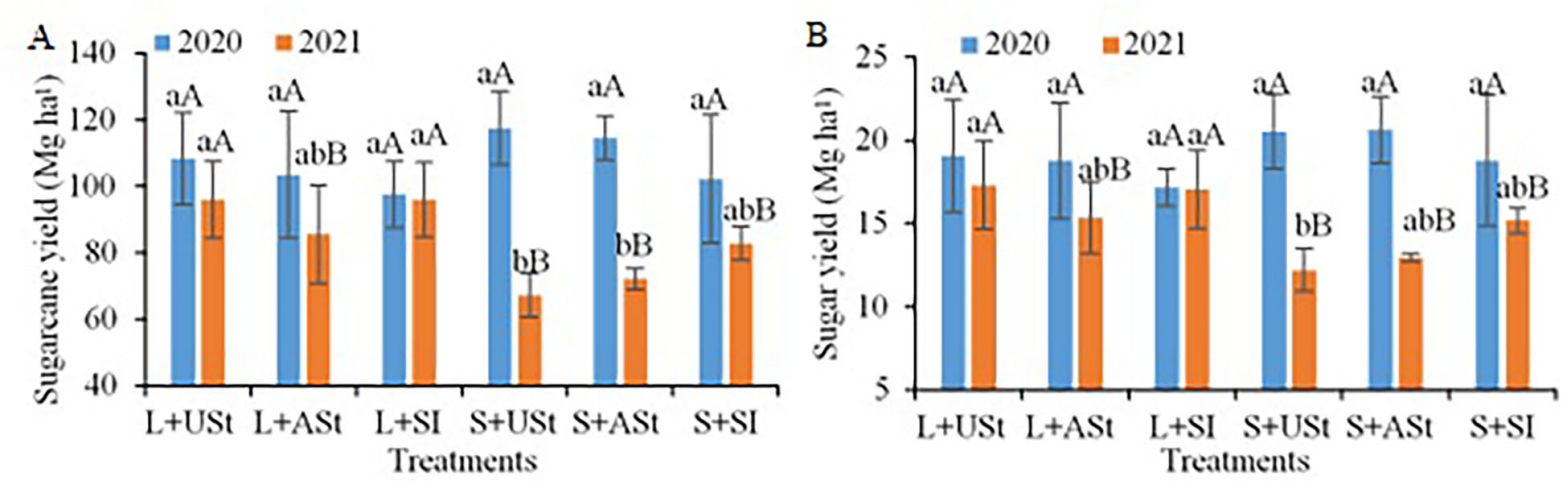
Decomposition of the interaction between treatments and crop seasons (2019/2020 and 2020/2021) for sugarcane yield **(A)** and sugar yield **(B)** in the site 2 (late-harvest site) as a function of the fertilizer source and application method. Lowercase letters compare treatments for each crop season and uppercase letters compare crop seasons for each treatment. L, Liquid fertilizer; S, Solid fertilizer; ASt, Application above straw; USt, Application under straw; SI, Application incorporated into the soil.

In the decomposition of the interaction between treatments and crop seasons for stalk yield, no differences were observed between treatments in the first crop season (**Figure 7A**). In the second crop season, treatments with the application of liquid fertilizers stood out for the highest values, especially with the USt and SI application methods. In addition, except for the treatments with USt and SI application methods of liquid fertilizer, all treatments had lower yields in the second crop season. In the decomposition of the interaction between treatments and crop seasons for sugar yield (**Figure 7B**), the difference between treatments was similar to that observed for stalk yield; that is, no differences were found between treatments in the first crop season. In the second crop season, treatments with liquid fertilizer had the highest values, mainly with the USt and SI application methods. Only treatments with USt and SI application methods applying liquid fertilizer did not reduce the sugar yield from the first to the second crop season.

**Figure 8:**
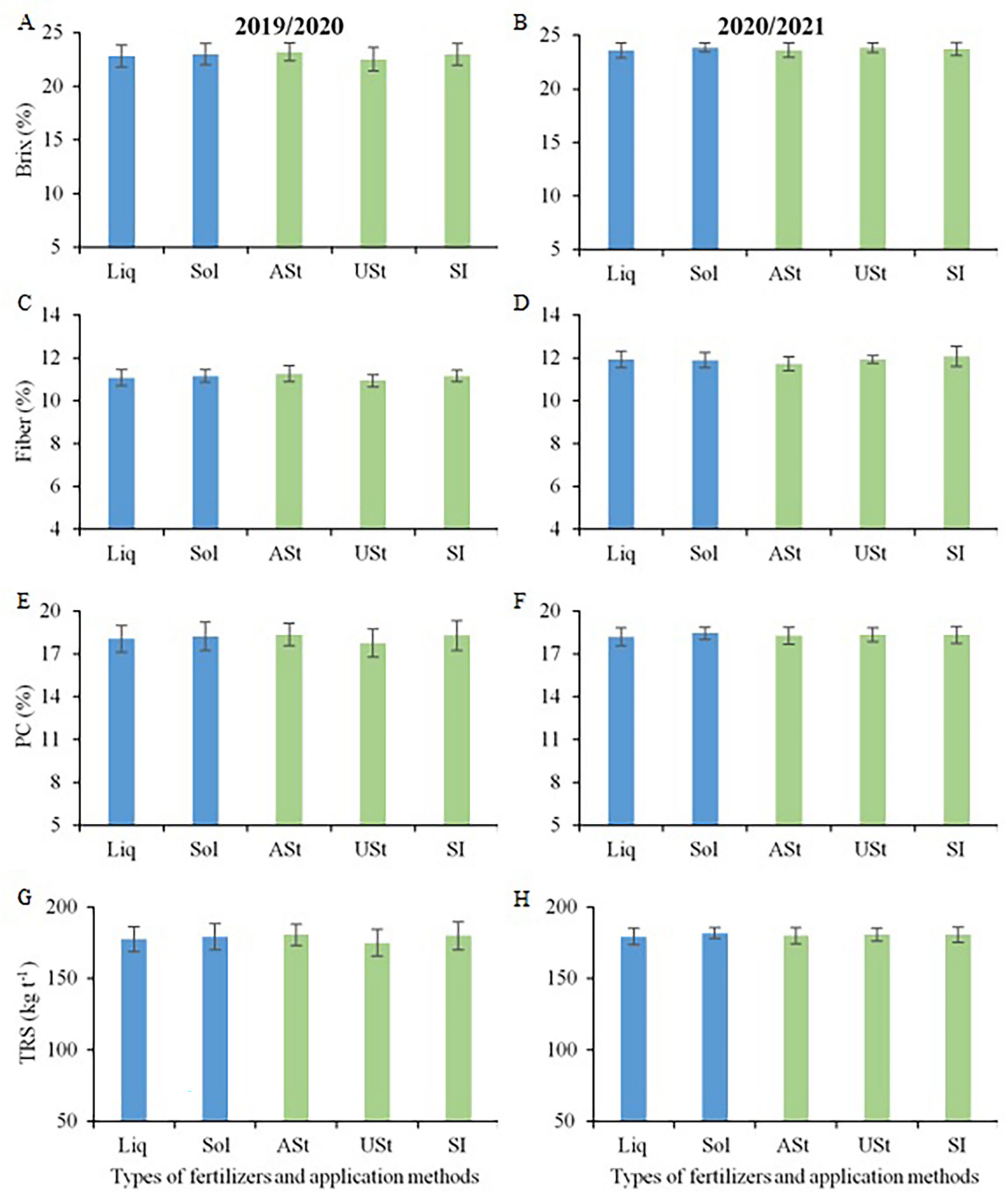
Comparison of means for Brix **(A, B)**, Fiber content **(C, D)**, Pol cane – PC **(E, F)** and total recoverable sugar (TRS, **G, H**) of sugarcane in the site 2 (late-harvest site) as a function of the fertilizer source and application method. Liq, Liquid fertilizer; Sol, Solid fertilizer; ASt, Application above straw; USt, Application under straw; SI, Application incorporated into the soil.

No technological variable was influenced by the study factors for the late harvest site of sugarcane (**Figure 8**). In the two crop seasons, Brix values ranged from 22.8 to 23.9%, fiber from 10.9 to 12.1%, PC from 17.7 to 18.4%, and TRS from 175 to 182 kg TRS Mg^−1^ of stalk.

**Figure 9:**
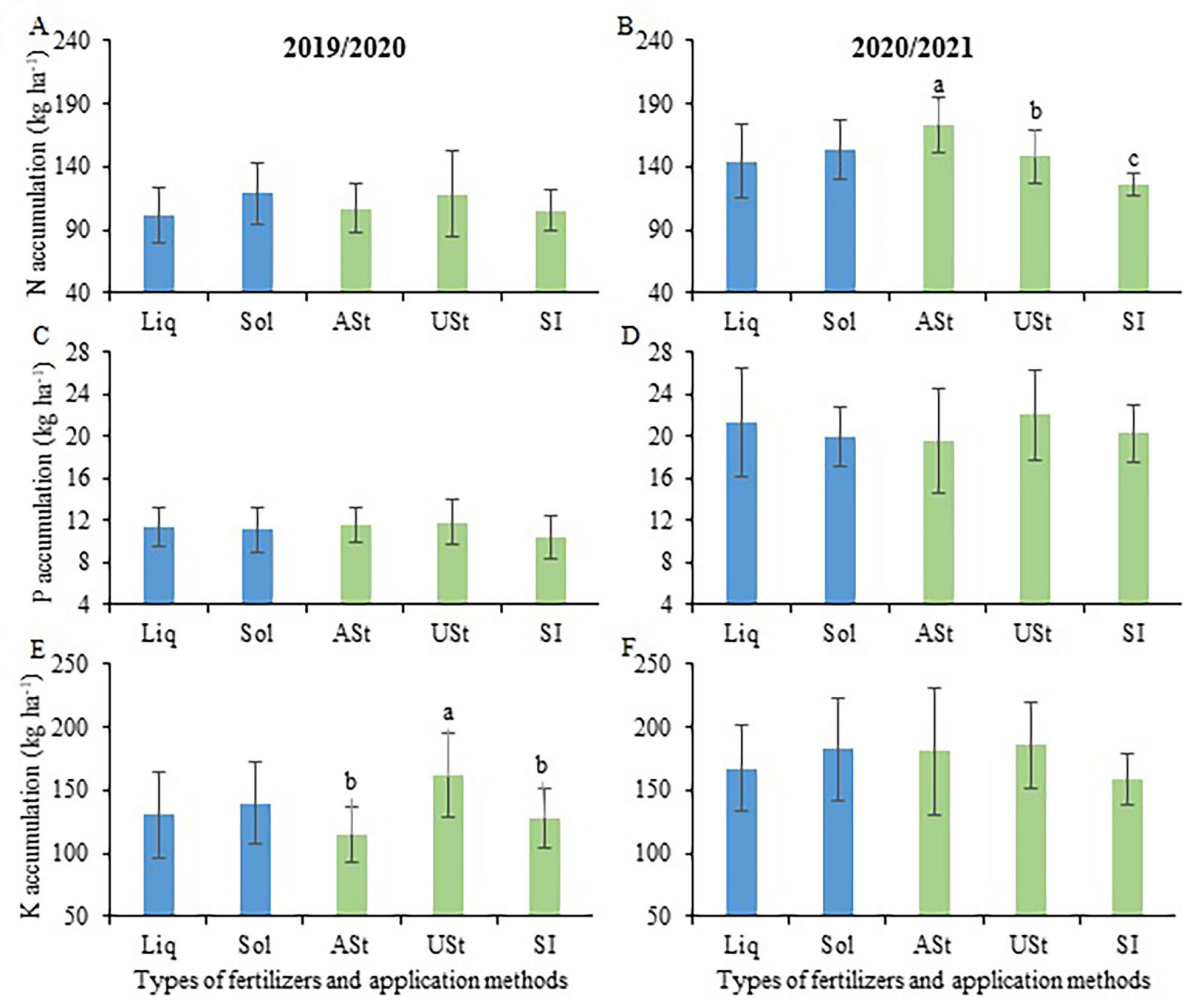
Comparison of means for the total accumulation of N **(A, B)**, P **(C, D)** and K **(E, F)** of sugarcane in the site 2 (late-harvest site) as a function of the fertilizer source and application method. Liq, Liquid fertilizer; Sol, Solid fertilizer; ASt, Application above straw; USt, Application under straw; SI, Application incorporated into the soil. Lowercase letters compare fertilizer application methods.

The nutrient levels (N-P-K) obtained in each plant tissue (i.e., stalk, tops, and dry leaves) are shown in **Supplementary Table 4**. There were no differences between treatments within each crop season. Comparing crop seasons, nutrient levels were higher in 2020/2021, except for the N content in dry leaves. In the second crop season, the ASt application method, regardless of fertilizer source, promoted the highest N accumulation (**Figures 9A, B**), followed by the USt and SI application methods. For P (**Figures 9C, D**), no differences were observed between the study factors, with accumulation values ranging from 10.4 to 21.3 kg ha^−1^ in the two crop seasons. For K (**Figures 9E, F**), the USt application method promoted the highest accumulation compared to the other application methods in the first crop season (+32%) with no differences in the second crop season (**Figure 9E**).

## 4 Discussion

### 4.1 Sugarcane yield

The fertilizer source and application method interacted with each other and resulted in specific fertilization recommendations for sugarcane over the sugarcane harvest season in south-central Brazil. At the site harvested at the beginning of the sugarcane harvest season (site 1; **Figure 3**), the highest yields were obtained with the use of liquid fertilizer incorporated into the soil in the middle of the sugarcane row (SI) and with solid fertilizer under sugarcane straw (USt), regardless of crop season. However, at the site harvested at the end of the sugarcane harvest season (site 2; **Figure 6**), these results depended on weather conditions close to the fertilization period. In the crop season with higher rainfall, the fertilizer source and application method did not interfere with sugarcane yield, while in the drier crop season, the delayed occurrence of rain in the spring reduced the existing water deficit during the winter season, and liquid fertilizer promoted better results, regardless of the application method.

At the early harvest site, fertilization in the two crop seasons was carried out in July. In this period, the weather conditions in the study region are characterized by scarce rainfall, mild temperatures, and a high water deficit (Alvares et al., 2013), which extends until mid-October/November (**Figure 2**). These conditions limit the growth of sugarcane and nutrient availability from the solid fertilizer to the crop. This factor is further aggravated by fertilizer applications onto the straw since the lack of heavy rainfall in this period prevents the contact of the fertilizer with the soil (Otto et al., 2016). This can be confirmed by the results obtained because, in general, the application method above sugarcane straw (ASt) led to the lowest sugarcane yield, regardless of fertilizer source.

The thick layer of straw onto the soil surface in sugarcane ratoon sites with a mechanized harvest exceeds 15 Mg ha^−1^ of dry matter (Aquino et al., 2018; Carvalho et al., 2019), demonstrating the capacity of this material to act as a physical barrier. Recently, studies have indicated that the partial removal of this straw from soil can promote agronomic benefits for the production system (Carvalho et al., 2017; Aquino et al., 2018; Carvalho et al., 2019). According to these studies, the removal of approximately 50% of the total straw on the soil improves the conditions for sugarcane sprouting, reduces the infestation of some pests, and has economic benefits through the processing of this residue. This management can also assist in fertilization programs carried out in sugarcane ratoons, reducing the physical barrier of contact of fertilizers with soil, especially in sites harvested at the beginning of the sugarcane harvest season. Furthermore, technologies where it is possible to supply fertilizers near the plant root system are a good alternative to maximize yield gains, as described by the SI application using liquid fertilizer in our research (**Figures 3C, D**). The higher yield promoted by liquid fertilizer is possibly associated with the immediate availability of nutrients to sugarcane plants due to its liquid form, favoring their uptake by the roots in the soil solution; conversely, when using solid fertilizer, sufficient moisture is required to dissolve the granules and subsequently make nutrients available in the soil solution (Taiz and Zeiger, 2009; Castro et al., 2022).

In a harvest period with low water availability, e.g., at the early period of the harvest season), the application of liquid fertilizers assists in the initial growth of sugarcane plants, especially in the sprouting process, due to the immediate availability of nutrients for root uptake (Silva et al., 2017). This, associated with the beneficial effect of straw in maintaining a certain level of soil moisture, contributes to a growth potential higher than that verified for solid fertilizer in the same application method (**Figures 3C, D**). In addition, the high rate of solid fertilizer in this application method (105 kg K_2_O ha^−1^, i.e., 87 kg K ha^−1^) causes some harmful effects on sugarcane due to the salt index of the fertilizer. Because of the lack of moisture in the soil, the nutrients from the solid fertilizer are not released (Castro et al., 2022) and a long-term effect of salt concentration occurs, which may affect the crop.

A high K_2_O application rate in a sugarcane ratoon can reduce crop yield. Almeida et al. (2015) observed that the highest yield of sugarcane ratoon harvested during the early period of the sugarcane harvest season in different soils, *Latossolo* (Oxisol) and *Argissolo* (Ultisol), was obtained with K_2_O rates of 117 (similar to the rate used in the present study) and 123 kg K_2_O ha^−1^, respectively, with a reduction in yield after these rates. These results demonstrate the harmful effect of high fertilizer rates, especially potassium fertilizer, on sugarcane ratoon yield. In the study conducted by Almeida et al. (2015), the surface application may have mitigated the salt effect of fertilizer on sugarcane, preventing direct contact with its root system. Similar results were reported by Flores et al. (2014), who observed that sugarcane yield in a *Latossolo* (Oxisol) decreased after 120 kg K_2_O ha^−1^.

In addition to the fertilizer source and application method, the time required for fertilizer application is essential for sugarcane. The timing of fertilizer application must coincide with favorable conditions for nutrient uptake by sugarcane (Castro et al., 2019; Congreves et al., 2021). Castro et al., (2022) found that the ideal time for N application in sugarcane ratoons can increase crop yield by up to 26 Mg ha^−1^. The authors also verified that the synchronization of the best moment for N application is more important for early and mid-season harvest periods, while at the late period of the sugarcane harvest season, this definition only slightly affected the sugarcane yield. For the early and middle periods of the sugarcane harvest season, the best period for fertilizer application ranges from 0 to 90 days after harvest (Castro et al., 2022). These results confirm those obtained in the present study, demonstrating the importance of specific fertilization management for sugarcane ratoon as a function of the harvest time of the site, increasing the yield and sustainability of the production system.

For fertilization in sites harvested during the late period of the sugarcane harvest season, the application methods interfered little with sugarcane yield, while the fertilizer source, depending on the weather conditions of the crop season, can directly interfere in sugarcane biomass production. In the south-central region of Brazil, weather conditions are different from those found in the early and middle periods of the sugarcane harvest season (Su et al., 2012; Otto et al., 2016; Corrêa et al., 2019). The highest levels of rainfall start in spring (October) along with favorable conditions for the vegetative growth of sugarcane, such as high temperatures (>25°C). Thus, fertilization in late harvest sites coincides with a period of high rainfall and favorable conditions for the release of nutrients from fertilizers (high moisture) and uptake by sugarcane (Otto et al., 2016; Franco et al., 2017). Unlike at the early harvest site, fertilization above sugarcane straw (ASt; **Figure 6**) did not differ from the other application methods, regardless of the fertilizer source, since the most frequent and intense rains (**Figure 2**) transported the fertilizer through the straw so that it reached the soil surface and was infiltrated in to the soil to be uptaken by the plant (Bowless et al., 2018). However, depending on the weather conditions of the crop season, the fertilizer source can interfere with crop yield, for example, when long periods of high temperature and absence of rainfall occur during the period favorable to plant growth in the summer season (Castro et al., 2017; Castro et al., 2022).

In crop seasons in which the weather phenomenon La Niña occurs in late winter and spring, rains in the southeast region of Brazil may be scarcer and start later (Andreoli et al., 2019). This phenomenon occurred in 2020 and was verified by the rainfall data, as the rainfall levels in November were much lower than those in the same period of 2019, resulting in a more severe and longer water deficit (**Figure 2**). The use of liquid fertilizers in this situation favored the initial growth of sugarcane, given the immediate availability of nutrients from this source associated with rainfall, which despite being scarcer favored faster sugarcane growth that resulted in a final yield of 25% higher than that observed with solid fertilizer.

Castro et al. (2017) observed that, depending on the crop season, sugarcane yield was similar when N fertilizer was applied to the surface or incorporated into the soil. This similarity occurred in a crop season in which the water deficit before fertilization was low. However, in general, the authors found that the incorporation of fertilizer at a depth of 0.08 m on both sides of the sugarcane row promoted yield gains of 13% compared to the surface application.

### 4.2 Sugarcane technological quality

None of the treatments interfered with the technological quality of sugarcane. This effect has also been reported in other studies that have evaluated the nutrition of sugarcane (Prado and Pancelli, 2008; Castro et al., 2017; Borges et al., 2019). In this context, the choice of liquid or solid fertilizer does not interfere with the technological quality of the raw material (**Figure 5**) at sites of south-central Brazil, although there are reports in the scientific literature showing that the adoption of liquid fertilizer may promote increments in the quality of the raw material (Gutiérrez-Miceli et al., 2017).

The higher sugar yield in some treatments was due to the higher stalk yield (Boschiero et al., 2020), as this variable is considered in the calculation of sugar yield (Fernandes, 2003). Despite the absence of differences between treatments for each site, the site harvested at the late period of the sugarcane harvest season had higher values of Brix, PC, and TRS when compared to the site harvested at the early period of the sugarcane harvest season. This occurred because this site was harvested after sugarcane underwent a longer period of low temperatures and water deficits (**Figure 2**). According to Cardozo et al. (2015), the more intense the water deficit and the lower the temperatures, the more intense the crop maturation process.

The higher technological quality in the second crop season for both sites, especially in terms of Brix, can be explained by the greater water deficit (**Figure 2**), intensifying sugarcane maturation. The values of variables related to technological quality were above the levels recommended for sugarcane, with Brix above 18°, fiber between 10.5 and 12.5%, and PC above 13% (Fernandes, 2003; Consecana, 2006; Dalri et al., 2021).

### 4.3 NPK accumulation by sugarcane

In our study, the accumulation of N, P, and K by sugarcane was not influenced by the fertilizer source or application method (**Figure 6**); that is, through either liquid or solid fertilization, sugarcane obtained the nutrients needed to express its development and reach the best levels of yield (Bautista et al., 2019; Boschiero et al., 2020). The nutrient content (**Supplementary Tables 1, 4**) differed slightly between the treatments. The differences occurred especially among crop seasons, where, overall, the 2020/2021 crop season presented the highest values. Moreover, having security when deciding on liquid or solid fertilization in sugarcane nutrition at times when there is insecurity in the world supply of fertilizers (Kennes et al., 2022) allows the farmer to have more alternatives to carry out appropriate nutritional management for the crop.

At the site harvested during the early period of the sugarcane harvest season, the higher accumulation for the USt application method, especially for N and P, was due to the higher stalk yield (Boschiero et al., 2020) promoted by this application method applying solid fertilizer. Among the crop seasons, the site harvested at the end of the sugarcane harvest season had higher NPK accumulation in the second crop season compared to the first. Though at this site, the stalk yield was lower in the second crop season, which can be justified by the higher nutrient content in sugarcane (**Supplementary Table 4**).

The NPK accumulation observed in the present study (**Figure 6**) was similar to that reported by Cherubin et al. (2019), who verified values of approximately 150 kg N ha^−1^, 18 kg P ha^−1^, and 270 kg K ha^−1^ in sites in the south-central region of Brazil. Under irrigated conditions, NPK accumulation by sugarcane can reach more than 300, 40, and 500 kg ha^−1^, respectively (Wanderley et al., 2021), i.e., values more than 100% higher than those observed here. This demonstrates the impact of a water deficit on the nutrient accumulation by sugarcane, as reported in this research, requiring specific fertilization management to reduce possible nutrient losses.

In a recent review considering 24 scientific papers, Otto et al. (2019) obtained average values of N, P, and K accumulation of 1.43, 0.53, and 2.09 kg Mg^−1,^ respectively, in the stalk. This result is similar to that obtained in the present study, in which at the site harvested during the early period of the sugarcane harvest season, the relative accumulation in the two crop seasons ranged from 1.17 to 1.34 kg N Mg^−1^ stalk, from 0.14 to 0.19 kg P Mg^−1^ stalk, and from 1.59 to 1.73 kg K Mg^−1^ stalk. For the site harvested during the late period of the sugarcane harvest season in the two crop seasons, the relative accumulation ranged from 1.23 to 1.98 kg N Mg^−1^ stalk, from 0.12 to 0.24 kg P Mg^−1^ stalk, and from 1.40 to 2.02 kg K Mg^−1^ stalk. These values of relative accumulation were similar to those observed in other sugarcane cultivars for N, P, and K (Silva et al., 2018; Castro et al., 2022), demonstrating that the accumulation of the macronutrients N, P, and K by the crop is similar to either liquid or solid fertilization regardless of the application method (i.e., above the straw [ASt], under the straw [USt], and incorporated into the soil in the sugarcane row [SI]).

## 5 Conclusion

The harvest period interferes with the definition of the best fertilization management for sugarcane. Additionally, throughout the sugarcane harvest season, there were differences between fertilizer sources (i.e., liquid or solid) and application methods (i.e., above or under straw layer on the soil, and into the soil). In the early period of the sugarcane harvest season, the fertilizer source and application method interacted with each other. In this situation, the highest stalk and sugar yields were obtained with the application of liquid fertilizer incorporated into the soil in the center of the crop row and solid fertilizer under the straw on the soil surface, generating increments of up to 33% in stalk yield compared to the other treatments. For the site where sugarcane was harvested in the late period, the liquid fertilizer promoted a 25% higher stalk yield compared to solid fertilizer in the crop season with low rainfall in the spring, while in crop season with normal rainfall, there were no differences between treatments. These results demonstrate the importance of the fertilizer source and application method based on harvest time in the recommendation of fertilization for sugarcane.

## Data availability statement

The raw data supporting the conclusions of this article will be made available by the authors, without undue reservation.

## Author contributions

Writing-original draft: SGQC, APC, and SAQC. Data curation: SGQC, and APC. Writing, revision and methodology: TRSC, RAC, and LBL. Formal analysis: SGQC, APC, SAQC, and TRSC. Project administration: SGQC, TRSC, and RAC. Resources: SGQC, SAQC, TRSC, and LBL. All authors contributed to the article and approved the submitted version.
