# Supplementary material for: Fertilizer source and application method influence sugarcane production and nutritional status
Source: Front Plant Sci. 2023 Mar 8;14:1099589. doi: 10.3389/fpls.2023.1099589 (PMC10032168; doi:10.3389/fpls.2023.1099589)
Supplement: Supplementary file 1 [file DataSheet_1.docx]

Supplementary Material

**
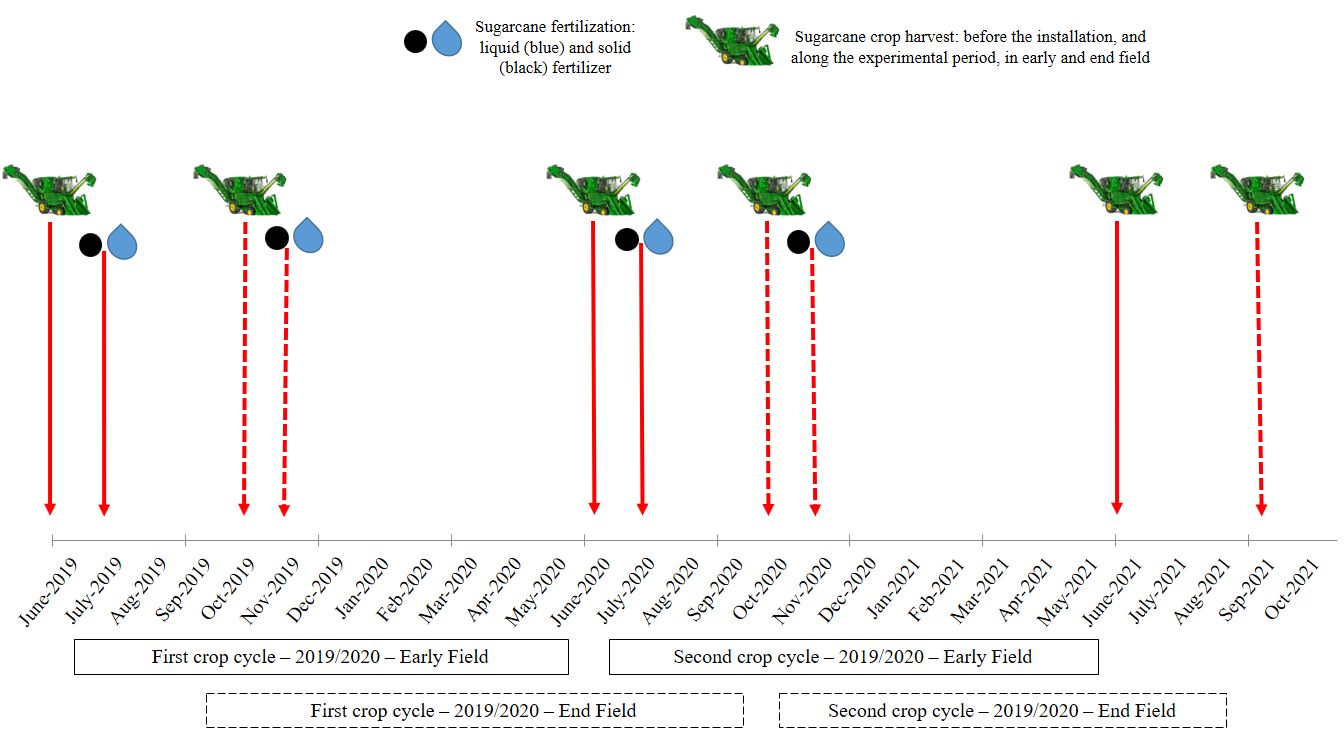
Supplementary Figure 1.** The experimental cronosequence adopted in early and end field during the experimental period. Continuous and doted arrows refer to early and end field, respectively.

**Supplementary Table 1**. Summary of the analysis variance for the nutrient content in stalk, dry leaves and tops of sugarcane in the site 1 (early-harvest site) as a function of fertilizer sources and application methods in the two crop seasons

| Source of variation | N stalk | P stalk | K stalk | N leaf | P leaf | K leaf | N tops | P tops | K tops |
| --- | --- | --- | --- | --- | --- | --- | --- | --- | --- |
|  | g kg^-1^ | | | | | | | | |
|  | --------------------------------------p-value - 2020-------------------------------------- | | | | | | | | |
| Fertilizer sources (F) | 0.89 | 0.46 | 0.27 | 0.54 | 0.26 | 0.94 | 0.84 | 0.26 | 0.33 |
| Application methods (MA) | 0.54 | 0.31 | 0.98 | 0.35 | 0.53 | 0.44 | 0.77 | 0.05 | 0.22 |
| F x MA | 0.63 | 0.43 | 0.56 | 0.33 | 0.71 | 0.29 | 0.84 | 0.29 | 0.05 |
| CV (%) | 14.9 | 20.7 | 32.3 | 20.5 | 23.8 | 61.3 | 15.4 | 13.3 | 14.2 |
|  | --------------------------------------p-value - 2021-------------------------------------- | | | | | | | | |
| Fertilizer sources (F) | 0.51 | 0.96 | 0.07 | 0.87 | 0.22 | 0.51 | 0.60 | 0.73 | 0.16 |
| Application methods (MA) | 0.12 | 0.06 | 0.87 | 0.71 | 0.001 | 0.21 | 0.98 | 0.07 | 0.22 |
| F x MA | 0.19 | 0.06 | 0.57 | 0.09 | 0.006 | 0.50 | 0.61 | 0.37 | 0.005 |
| CV (%) | 11.4 | 16.5 | 25.3 | 16.1 | 13.6 | 51.7 | 15.4 | 10.7 | 12.6 |
| Joint Analysis | | | | | | | | | |
| Highest RMS/Lowest RMS | 1.60 | 2.02 | 1.49 | 1.53 | 2.46 | 1.13 | 1.08 | 1.42 | 1.20 |
|  | p-value | | | | | | | | |
| Treatments (T) | 0.05 | 0.06 | 0.003 | 0.005 | 0.22 | 0.006 | 0.23 | 0.003 | <0.001 |
| Crops Seasons (Y) | 0.18 | 0.04 | 0.11 | 0.21 | 0.19 | 0.13 | 0.10 | 0.03 | 0.19 |
| T x Y | 0.91 | 0.68 | 0.99 | 0.98 | 0.15 | 0.98 | 0.98 | 0.95 | 0.96 |
| 2019/2020 | 2.10 | 0.26 a | 3.01 | 3.31 | 0.21 | 1.65 | 6.68 | 0.94 b | 14.53 |
| 2020/2021 | 2.17 | 0.23 b | 3.14 | 3.40 | 0.24 | 1.83 | 6.91 | 0.99 a | 14.91 |

CV – coefficient of variation

**Supplementary Table 2**. Test of means for P content in dry leaves and K content in sugarcane tops in the site 1 (early-harvest site)

| Fertilizer | P leaf (g kg^-1^) | | | Fertilizer | K tops (g kg^-1^) | | |
| --- | --- | --- | --- | --- | --- | --- | --- |
|  | ASt | USt | SI |  | ASt | USt | SI |
| Liquid | 0.23 aA | 0.27 aA | 0.24 bA | Liquid | 15.01 aA | 15.23 aA | 12.78 bA |
| Solid | 0.16 bB | 0.23 aA | 0.29 aA | Solid | 16.23 aA | 12.66 aB | 17.52 aA |

Sol: Solid; Liq: Liquid; ASt: above straw; USt: Under straw; SI: soil incorporated; equal lowercase letters in the column and uppercase letters in the row do not differ from each other by Tukey's test (p<0.05)

**Supplementary Table 3**. Test of means for nutrient content (joint analysis) in the site 1 (early-harvest site)

| Treatment | K stalk | N leaf | K leaf | P tops | K tops |
| --- | --- | --- | --- | --- | --- |
| Sol_ASt | 3.34 ab | 3.16 b | 1.91 ab | 1.07 a | 16.24 a |
| Sol_USt | 3.09 bc | 3.39 b | 1.62 b | 0.89 cd | 12.65 b |
| Sol_SI | 3.61 a | 3.36 b | 1.87 ab | 0.98 abc | 16.76 a |
| Liq_ASt | 2.67 c | 3.92 a | 1.15 b | 1.04 ab | 14.99 a |
| Liq_USt | 3.06 bc | 3.33 b | 1.41 b | 0.94 bcd | 15.19 a |
| Liq_SI | 2.67 c | 2.98 b | 2.49 a | 0.85 d | 12.49 b |

Sol: Solid fertilizer; Liq: Liquid fertilizer; ASt: above straw; USt: Under straw; SI: soil incorporated; equal lowercase letters in the column do not differ from each other by Tukey's test (p<0.05)

**Supplementary Table 4**. Summary of the analysis variance for the nutrient content in stalk, dry leaves and tops of sugarcane in the site 2 (late-harvest site) as a function of fertilizer sources and application methods in the two crop seasons

| Source of variation | N stalk | P stalk | K stalk | N leaf | P leaf | K leaf | N tops | P tops | K tops |
| --- | --- | --- | --- | --- | --- | --- | --- | --- | --- |
|  | g kg^-1^ | | | | | | | | |
|  | --------------------------------------p-value - 2020-------------------------------------- | | | | | | | | |
| Fertilizer sources (F) | 0.32 | 0.47 | 0.37 | 0.17 | 0.39 | 0.78 | 0.62 | 0.19 | 0.32 |
| Application methods (MA) | 0.64 | 0.38 | 0.62 | 0.99 | 0.84 | 0.62 | 0.20 | 0.90 | 0.15 |
| F x MA | 0.55 | 0.97 | 0.26 | 0.40 | 0.61 | 0.92 | 0.40 | 0.80 | 0.51 |
| CV (%) | 17.3 | 24.9 | 26.7 | 24.1 | 27.3 | 48.4 | 13.9 | 15.7 | 15.4 |
|  | --------------------------------------p-value - 2021-------------------------------------- | | | | | | | | |
| Fertilizer sources (F) | 0.38 | 0.40 | 0.97 | 0.74 | 0.27 | 0.54 | 0.07 | 0.31 | 0.88 |
| Application methods (MA) | 0.55 | 0.06 | 0.70 | 0.83 | 0.40 | 0.76 | 0.05 | 0.10 | 0.26 |
| F x MA | 0.68 | 0.36 | 0.30 | 0.09 | 0.47 | 0.07 | 0.63 | 0.20 | 0.67 |
| CV (%) | 22.7 | 17.3 | 29.4 | 6.5 | 74.9 | 7.4 | 17.4 | 13.7 | 16.4 |
| Joint Analysis | | | | | | | | | |
| Highest RMS/Lowest RMS | 3.41 | 2.98 | 6.89 | 5.38 | 6.48 | 2.19 | 4.68 | 2.56 | 1.72 |
|  | p-value | | | | | | | | |
| Treatments (T) | 0.54 | 0.82 | 0.06 | 0.53 | 0.50 | 0.19 | 0.44 | 0.72 | 0.89 |
| Crops Seasons (Y) | 0.001 | <0.001 | <0.001 | <0.001 | 0.002 | <0.001 | <0.001 | <0.001 | 0.01 |
| T x Y | 0.69 | 0.07 | 0.94 | 0.53 | 0.46 | 0.86 | 0.10 | 0.10 | 0.23 |
| 2019/2020 | 2.09 b | 0.23 b | 2.99 b | 3.14 a | 0.25 b | 2.28 b | 5.96 b | 0.78 b | 11.63 b |
| 2020/2021 | 2.93 a | 0.57 a | 7.12 a | 1.73 b | 2.39 a | 10.11 a | 10.32 a | 1.47 a | 14.35 a |

CV – coefficient of variation
